# Metropolitan Hospital Saturday Fund

**Published:** 1905-05-06

**Authors:** 


### METROPOLITAN HOSPITAL SATURDAY FUND.

The Lord Mayor presided at the meeting of the Hospital Saturday Fund which was held at the Mansion House on April 29th.

The report of the Council stated that the collection in 1904 was the highest on record, amounting to £24,344, and Mr. A. S. Harvey, the chairman of the Council, who presided, remarked that although the work of the Fund during 1904 was larger than ever before, the percentage of expenses was less, being under 10 per cent.

Medallions were presented to those who had attended the Fund's First Aid classes, and passed the final examination of the St. John's Ambulance Association. Some diplomas of merit for saving life were also awarded. Dr. Arthur Latham ave an address on the Industrial Classes and the Treatment f Tuberculosis, in which he briefly reviewed the present situation of tuberculosis and the means taken to combat it. He drew attention to the fact that the great consumption hospitals should do more than act as educational centres— and he pointed out that those in towns should act as receiving houses for patients to be drafted into country sanatoria. He said the matter was in the hands of the industrial classes, who could act through their friendly societies.

